# A debatable representation of all-cause mortality in Norway in 2024—authors’ reply

**DOI:** 10.1177/14034948251403426

**Published:** 2026-01-07

**Authors:** Richard Aubrey White, Anders B. Nygaard, Arne Søraas, Gunhild A. Nyborg

**Affiliations:** 1 Independent researcher, Oslo, Norway; 2 Department of Microbiology, Oslo University Hospital, Oslo, Norway

**Keywords:** COVID-19, all-cause mortality, excess mortality

We thank the editor for the opportunity to respond to the letter from Knudsen et al. [1] —nine authors from the Norwegian Institute of Public Health (NIPH)—who commented on our previous work [2] that drew different conclusions to NIPH.

Knudsen et al. assert that “the purpose of [NIPH’s] continuous surveillance of total mortality is to monitor and describe mortality trends in real time.” We agree their model is suitable for this purpose. However, it is not suitable for assessing whether the pre-pandemic decreasing trend in mortality has shifted. Until 2024, NIPH used a pre-pandemic baseline to assess the effects of the pandemic on all-cause mortality. In 2025, NIPH changed the model for 2024 to include a post-pandemic year—2023, which had substantial excess mortality. This resulted in a press release “Mortality back to the level before the pandemic,” in which NIPH combined results from models with pre-pandemic baselines (for 2022 and 2023) with their new model for 2024:

In 2022 there was excess mortality in the oldest age groups [model with pre-pandemic baseline]. In 2023 there was also excess mortality in the age groups under 40 years old [model with pre-pandemic baseline] . . . In 2024, NIPH did not find excess mortality for any age groups, including those under 40 years old [model that includes 2023 in the baseline] [3].

We have used [square brackets] to identify which model was used for which statements to show how switching baselines creates a misleading narrative.

This is problematic for two reasons. Firstly, these models have different purposes—evaluating long-term/pandemic effects versus real-time surveillance, and hence their results cannot be directly compared. Secondly, the model switch was not clearly labeled in NIPH’s results interpretation in the press release, misleading the reader.

This is not a neutral update but a modeling choice with predictable effects: it reduces comparability with pre-pandemic trends and makes “no statistically significant excess” more likely by construction. Readers should be shown how conclusions depend on this choice through transparent sensitivity analyses (e.g., baselines excluding 2023, and side-by-side reporting of absolute and standardized metrics). Absent such discussion, the headline inference may be mistaken for a data-driven finding when, in part, it reflects a baseline redefinition.

Knudsen et al. assert that their purpose is “not to evaluate . . . pandemic strategies,” yet by directly comparing two periods with vastly different pandemic strategies, their press release strongly implies that the current COVID-19 strategy is successful:

After periods with high mortality during the COVID-19 pandemic [model with pre-pandemic baseline], new estimates from NIPH show that mortality in Norway in 2024 was within expected levels [model that includes 2023 in the baseline] [3].

This comparison evaluates pandemic strategies, regardless of stated intent.

Knudsen et al. note that “annual totals can reveal longer-term trends in mortality.” We agree. Our model shows that the pre-pandemic mortality trend has broken, “suggest[ing] a new elevated mortality baseline and a reduction or reversal of Norway’s pre-pandemic mortality decline” [2]. NIPH, as custodian of the Cause of Death Registry, should be investigating this trend break.

Instead, Knudsen et al. focus on absolute values, obscuring this trend break: “In 2023 and 2024, the age-standardized mortality rate . . . returned to pre-pandemic levels.” This is technically accurate but substantively misleading: “return” means stagnation after five decades of improvement. What is causing this flattening? Notably absent is any discussion of what is driving these deaths or what mitigation measures might prevent them.

Knudsen et al. continue to ignore trends when discussing life expectancy: “recent reports suggest a [European] return toward pre-pandemic life expectancy levels.” However, accounting for 2010–2019 trends reveals continued deficit: life expectancy in 2024 was lower than forecast for 88% of EU countries, with an average deficit of 0.27 years [4]. Since, on average, EU life expectancy increased by 0.18 years annually from 2010–2019, it is now 1.5 years behind its pre-pandemic trajectory.

As widespread SARS-CoV-2 transmission persists into 2025, determining whether the pandemic and COVID-19 have altered mortality trends is essential—the primary aim of our study. Crucially, our model can detect these changes, while NIPH’s cannot.

We agree that using a pre-pandemic baseline increases the uncertainty of estimates for 2024. However, when evaluating the potential effects of a virus non-existent before 2019, that continues to reinfect large parts of the population annually, we believe there is no other choice.

With our model showing an excess of 5000 potentially preventable deaths in 2023 and 2024—including younger age groups experiencing statistically significant excess mortality—we would expect NIPH to initiate investigations. Rather than examining this trend break, Knudsen et al. insist that mortality has “returned to pre-pandemic levels.”
